# Sustainable Collagen Film Preparation with Tannins Extracted from Moroccan Pomegranate Byproduct Varieties: Thermal, Structural, and Nanoscaled Studies

**DOI:** 10.1021/acsomega.4c02321

**Published:** 2024-06-12

**Authors:** Sara El Moujahed, Faouzi Errachidi, Ana-Maria Morosanu, Hicham Abou Oualid, Sorin Marius Avramescu, Mihaela Dragoi Cudalbeanu, Fouad Ouazzani Chahdi, Youssef Kandri Rodi, Rodica-Mihaela Dinica

**Affiliations:** † Laboratory of Applied Organic Chemistry, Faculty of Sciences and Technologies, Sidi Mohamed Ben Abdellah University, Fez 30050, Morocco; ‡ Laboratory of Functional Ecology and Engineering Environment, Faculty of Sciences and Technologies, Sidi Mohamed Ben Abdellah University, Fez 30050, Morocco; § Institute of Biology Bucharest, Romanian Academy, Bucharest 060031, Romania; ∥ Green Energy Park, IRESEN-UM6P, Benguerir 43150, Morocco; ⊥ Department of Organic Chemistry, Biochemistry and Catalysis, Faculty of Chemistry, University of Bucharest, Bucharest 050663, Romania; # Faculty of Land Reclamation and Environmental Engineering, University of Agronomic Sciences and Veterinary Medicine of Bucharest, Bucharest 011464, Romania; ¶ Laboratory of Organic Chemistry, Faculty of Sciences and Environment, Dunarea de Jos University of Galati, Galati 800008, Romania

## Abstract

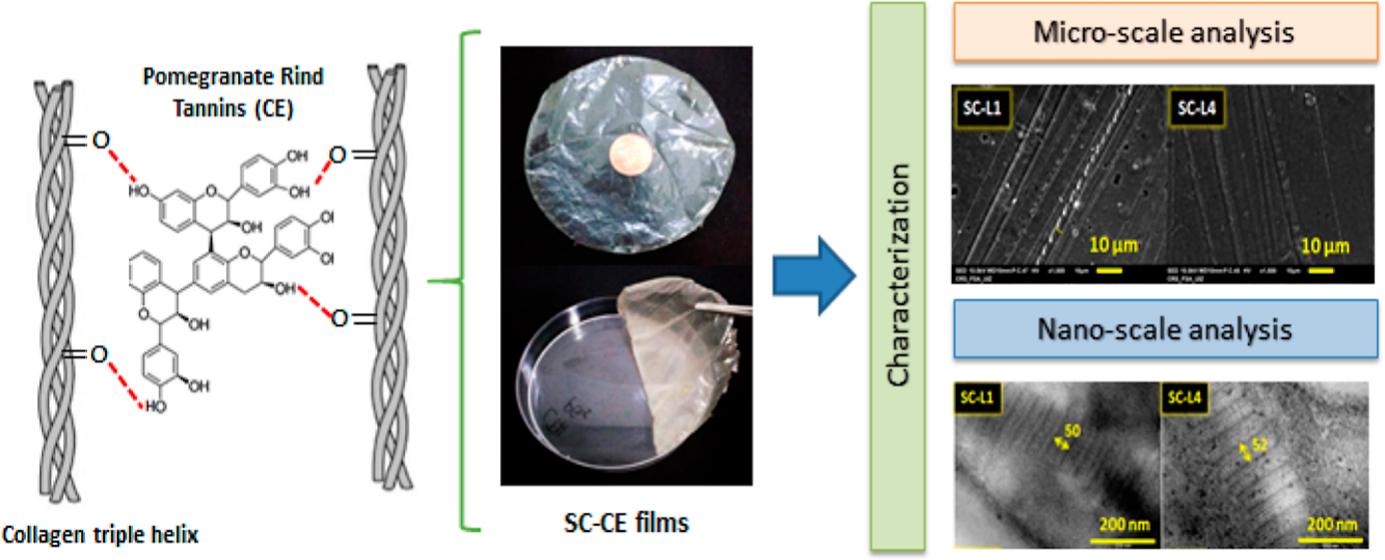

Recently, obtaining collagen films using a cross-linking technique has been a successful strategy. The current investigation used six cross-linker extracts (CE) from six different pomegranate varieties’ byproducts to make and characterize collagen-tannin films using acid-soluble collagen (SC). The polymeric film has a yellow hue after CE incorporation. Fourier transform infrared spectroscopy assessed the impact of CE and its successful interaction within the matrix. The shifts verify different interactions between extracts and collagen functional groups, where they likely form new hydrogen bonds, retaining their helix structure without damaging the matrix. Scanning electron microscopy was used to analyze the morphology and fiber size. The average diameter of the fibers was found to be about 3.64 μm. Thermal behaviors (denaturation and degradation) were investigated by thermogravimetric analysis. The weight losses of cross-linked films increased by around 20% compared to non-cross-linked ones. This phenomenon was explained by the absence of telopeptide sections in the collagen helical structure, typically reinforced by lysine and hydroxylysine covalent linkages. Nanoscaled observations were also accomplished using transmission electron microscopy (TEM) on SC and SC-CE. The TEM analysis confirmed the CE polymerization degree effect on the cross-linking density via the overlap sequences, ranging up to 32.38 ± 2.37 nm on the fibril. The prepared biodegradable collagen-tannin film showed higher cross-linking density, which is expected to improve the biomaterial applications of collagen films while exploiting the underrated pomegranate byproducts.

## 1 Introduction

Eco-friendly polymer-based multifunctional biomaterials have created a new wave in food, biomedical, and other sectors.^1,2^ In the food sector, collagen-based materials are starting to get a lot of attention; in addition to their use in nutraceuticals as biopeptides,^3^ they are included as a texture modifier^4^ and in edible packaging.^5^ In the medical sector, collagen is the most often utilized biopolymer in regenerative medicine. Due to its excellent biocompatibility with tissues and ability to resorb after implantation, it has found wide usage.^6^

Collagen has been detected in two forms: atelocollagen (acid-soluble) and telocollagen (insoluble). The two forms have essentially the same amino acid composition, FTIR vibrational spectra, and X-ray diffraction pattern^1^ (Figures S1 and S2 in Supporting Information), but their solubility has only a biological significance as it reflects their distinct roles in biomedical applications.^7,8^ Our prior studies focused on telocollagen, which was limited to tanning applications.^1,9^ The present study advances the field by using atelocollagen type I, labeled SC, which offers greater homogeneity and control, particularly in film formulation for diverse applications. Indeed, as a medical biomaterial, this collagen with cleaved extensor peptides finds relevance for regenerative medicine and tissue engineering.^6^

Collagen is mostly investigated in film form as a tissue regeneration material and protective coating.^10^ In addition, collagen is an excellent biopolymer to use when combined with another polymer and/or biopolymer.^11^ Collagen-based combinations with other hydrophilic polymers can be utilized to create high-quality films.^12,13^ So, it is necessary to modify collagen materials with various additives to increase their stability and functionalities. On top of that, nontoxic additives are needed.

The stabilization of collagen can be caused by cross-linking with tannins.^14−16^ Tannin compounds may function as cross-linkers for collagen owing to the carboxyl and hydroxyl groups present in their structure.^2^ They can interact with amino and carboxylic hydrophilic functional groups from the collagen polymeric chain through hydrogen bonds.^9,17^ By doing so, they could enhance their physicochemical characteristics. The primary commercial sources of vegetable tannins are *Caesalpinia spinosa* (tara), *Acacia mearnsii* (wattle), *Schinopsis balansae* (quebracho), *Terminalia chebula* (myrobalan), *Castanea vesca* (chestnut), and *Rhus coriaria* (sumac).^18,19^ Because they are expensive, vegetable tannins have not been utilized extensively in Morocco, particularly on an industrial basis.

On the other hand, vegetable tannins are extracted from wood, leaves, rinds, fruits, and beneficially from fruit byproducts and are safe for humans.^20^ The valorization of fruit byproducts holds critical importance in contributing to sustainability efforts. Tannins are the distinctive quality of pomegranate (*Punica granatum* L.) byproducts: they represent up to 28% of the rind.^21^ Pomegranate rind (PR) tannins have multifunctional properties such as antioxidant, anti-inflammatory, antidiabetic, anticancer, antiviral, and antimicrobial properties,^22−24^ which are valuable for developing pharmaceuticals and medical devices. Nonetheless, almost all of the pomegranate rind is discarded during industrial manufacturing.

Pomegranate rind (PR) extract has been used in biopolymeric matrices in several investigations to create packaging or active films such as collagen-chitosan films modified with pomegranate polyphenols using ultrasound to enhance the endurance of film psychological stress. Cross-linking has shown great improvement in mechanical, antioxidant, and antibacterial properties.^17,25,26^ Likewise, Costa et al.^27^ prepared polymeric films containing pomegranate rind extract based on PVA/starch/PAA blends, which showed in vitro antibacterial and healing activity. In addition, for the topical treatment of candidiasis, polymeric films containing pomegranate rind extract can serve as a drug delivery platform.^28^ In food applications, Vargas-Torrico et al.^29^ manufactured gelatin and carboxymethylcellulose-based films cross-linked to pomegranate rind (PR) extract with plasticizing effect and antioxidant, UV-light barrier, and pH-sensitive properties for preserving raspberries. Noncytotoxicity is also an important essential for biofilms. Previous reports confirmed that active collagen films with pomegranate peel extract are noncytotoxic packing materials.^30,31^

To the best of our knowledge, there is no report about the modification of collagen films with Moroccan pomegranate varieties’ byproducts, which are estimated to be more than 53,000 tons of rinds (between 40 and 50% of the total fruit’s weight) wasted per year.^32,33^ In this context, research has been focused on developing collagen films for food and medical applications by combining collagen with tannins extracted from underexploited byproducts of six pomegranate varieties (Djeibi, Mersi, Sefri 1, Sefri 2, Mollar de Elche, and Hicaz) with global commercial interest in Morocco. The purified cross-linked films (SC-CE) were characterized by studying their structural and thermal properties. Morphology and nanoscaled observations were made using SEM and TEM analysis. FTIR analyses were done to characterize cross-linked functional groups, and TGA was used to measure the weight loss of each SC-CE film.

## 2 Materials and Methods

### 2.1 Chemicals and Instruments

All reagents and chemicals were of analytical grade and used without further purification, except where explicitly specified. Acid Soluble Collagen type I (SC) from bovine was purchased from Sigma-Aldrich. Cross-linker solutions were lyophilized in a freeze-dryer [Christ Alpha 1–4 LD plus (Martin Christ, Germany)].

### 2.2 Raw Materials

**Figure 1:**
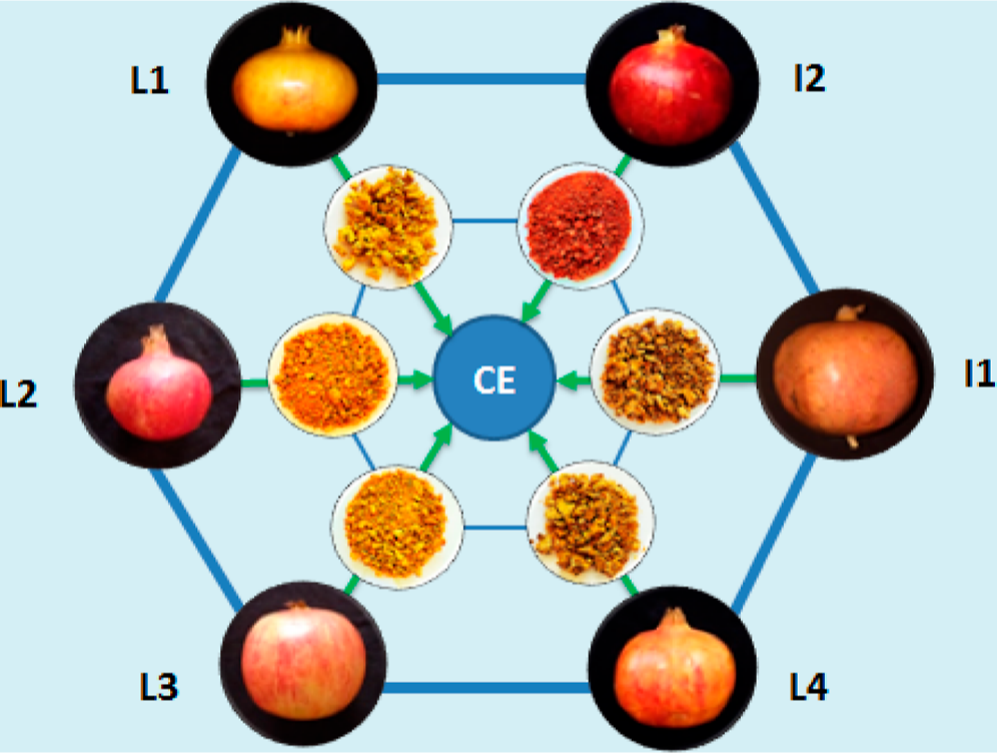
Studied commercial pomegranate cultivars [Djeibi (L1), Mersi (L2), Sefri 1 (L3), Sefri 2 (L4), Mollar de Elche (I1), and Hicaz (I2)] and their rind fractions used to produce the cross-linker extract (CE).

Byproducts from six pomegranate (*P. granatum* L.) cultivars, used by Moroccan agri-food industries for juice production, were collected at the same maturity level in October 2021. The cultivars comprised four local varieties [Djeibi (L1), Mersi (L2), Sefri 1 (L3), and Sefri 2 (L4)] and two imported varieties (Mollar de Elche (I1) and Hicaz (I2); Figure 1). Prior characterizations by our lab^33^ were done for their pomological, organoleptic, biochemical, and chemical traits. The rinds were manually peeled, shade-dried at ambient temperature, automatically ground, and hermetically stored at 4 °C for analysis. Each analysis was performed in triplicate.

### 2.3 Preparation Procedure

#### 2.3.1 Preparation of Cross-Linker Extract Solutions

Cross-linker extracts were carried out using the method reported in a previous work.^33^ Pomegranate rinds (PRs) were extracted using an aqueous-based solid–liquid method with heated ultrapure water at 20 °C, protected from light. The resulting powdered extracts, labeled L1, L2, L3, L4, I1, and I2, were dissolved in ultrapure water to make 0.1% (w/v) cross-linker solutions (CE). In the subsequent experiments, the freshly prepared CE solutions had a pH range of 3–3.5. Chemical compositions and molecular weight distributions of CE solutions were characterized previously by HPLC-DAD and SEC (Figures S3 and S4, and Table S1 in Supporting Information).

#### 2.3.2 Cross-Linked Film Preparation

Films were prepared by using solution casting and characterized for their thermal properties and structure. Six different collagen-based films (SC-CE) were prepared by drying a suspension of SC fibers on Petri plates. Films were prepared from a soluble collagen powder (control) and collagen cross-linked to phenolic compounds (CE) from PR extracts.

Briefly, a suspension containing 0.5% (w/v) collagen was swollen overnight in 0.05 M acetic acid at 4 ± 2 °C. The resulting suspension was added (ration 1:1) to CE solutions at 0.1% (w/v) and coded as SC-L1, SC-L2, SC-L3, SC-L4, SC-I1, and SC-I2 respectively. Samples were shaken for 1 h at pH 3 under a speed of 650 rpm. Subsequently, the mixture was placed under mild ultrasonic (40 kHz, 120 W) for about 1 min to remove air bubbles and then several drops of ethanol were added to eliminate air bubbles further.^34^ The SC-CE films with a thickness of around 0.03 mm were formed by casting the solution on Petri plates with a diameter size of 9.2 cm. The cross-linked films were allowed to dry overnight under an extractor hood at 20 °C, with adequate ventilation.^35^

### 2.4 Characterization Techniques

#### 2.4.1 Vibrational Studies by FTIR Analysis

Fourier transform infrared (FTIR) spectroscopy measurements were taken using a Thermo Scientific Nicolet iS10 spectrometer equipped with an ATR accessory in the 400–4000 cm^–1^ range. Basically, this method is based on the principle that molecular bonds in any material absorb specific frequencies of infrared light, causing a vibration. The resulting absorption is characteristic of a molecular fingerprint. The spectrum contains many peaks; each peak corresponds to different vibrational modes, such as stretching and bending. These peaks are analyzed to identify functional groups and then the entire structure.

#### 2.4.2 Thermogravimetric Analysis Measurement

All SC films (treated with L1, L2, L3, L4, I1, and I2 PR extracts) were subjected to thermogravimetric analysis conducted using TGA from NETZSCH STA 449 F1 Jupiter to characterize the overall thermal denaturation/degradation events. Samples (about 10 mg) were analyzed over the temperature range between 30 and 500 °C at a 10 °C/min heating rate under an N_2_ atm flow (60 mL/min).

#### 2.4.3 Electron Microscopy and Elemental Composition

##### 2.4.3.1 Scanning Electron Microscopy

Scanning electron microscopy (SEM) images were captured using a JEOL JSM-IT 100 microscope, incorporating an energy-dispersive X-ray spectroscopy (EDS) microanalyzer, operating at 120 kV. The analysis achieved a maximum resolution of approximately 100 nm. ImageJ software was utilized for accurate measurement of the SC fiber dimensions.

##### 2.4.3.2 Transmission Electron Microscopy

In order to perform TEM analyses on collagen films cross-linked to CE from L1, L2, L3, L4, I1, and I2 aqueous extracts, we processed the material in two variants: large fragments of collagen film subjected to the entire protocol and fragmented at the end of it and small fragments of collagen processed directly. Small fragments of the collagen films were prefixed overnight in 4% glutaraldehyde in 0.05 M cacodylate buffer pH 7.4 at 4 °C, and then they were further processed according to the routine protocol.^36^ After six successive washes with 0.05 M cacodylate buffer, the samples were postfixed with 4% OsO_4_ (in 0.1 M cacodylate buffer) for 2 h at room temperature, followed by the other six washes, and then dehydrated in a graded series of ethanol (30, 50, 70, 90, and 100%). After treatment with propylene oxide (PO) and pre-embedment of samples (PO and Epon mix), the final embedment at 60 °C for at least 24 h followed. Sectioning was performed on the Ultrotome III LKB ultramicrotome with a glass knife (70–90 nm thick), and the ultrafine sections were double counterstained with Uranyless and lead citrate. All the prepared grid samples were analyzed using a JEM-1400 (JEOL) operated at 80 kV accelerating voltage and visualized with a Quemesa CCD camera (Olympus Soft Imaging Solutions).

## 3 Results and Discussion

### 3.1 Optical Observations

**Figure 2:**
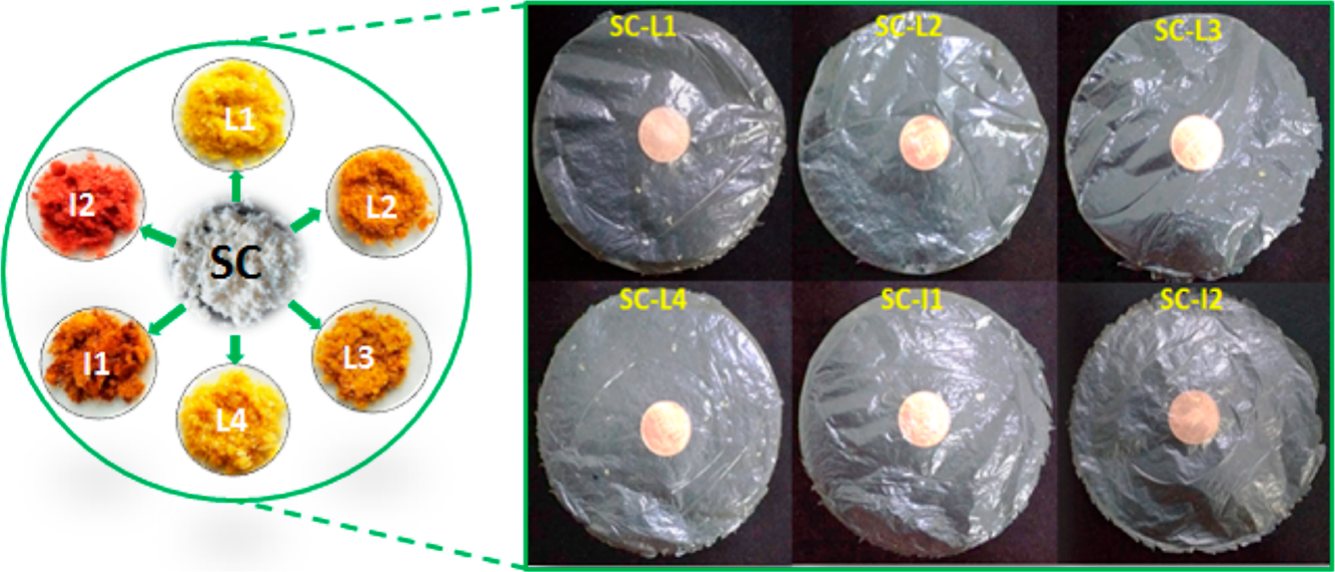
Schematic presentation of cross-linked SC with extracts from PR varieties (L1, L2, L3, L4, I1, and I2) and optical images of SC-L1, SC-L2, SC-L3, SC-L4, SC-I1, and SC-I2 films.

The obtained extracts illustrated in Figure 2 were previously characterized for their phenolic compositions and molecular size distributions (Figures S3 and S4 in Supporting Information). The results showed that the molecular weight distributions of their phenolic compounds were based on high molecular weight condensed tannins formed by the polymerization of the catechin monomer.^33^

On the other hand, to develop a collagen film with desired properties, it is necessary to choose a suitable collagen that can easily be used as a raw material for extruding or casting of collagen-based films. Hence, not all collagen extraction methods result in a collagen product that is suitable for film preparation.

**Figure 3:**
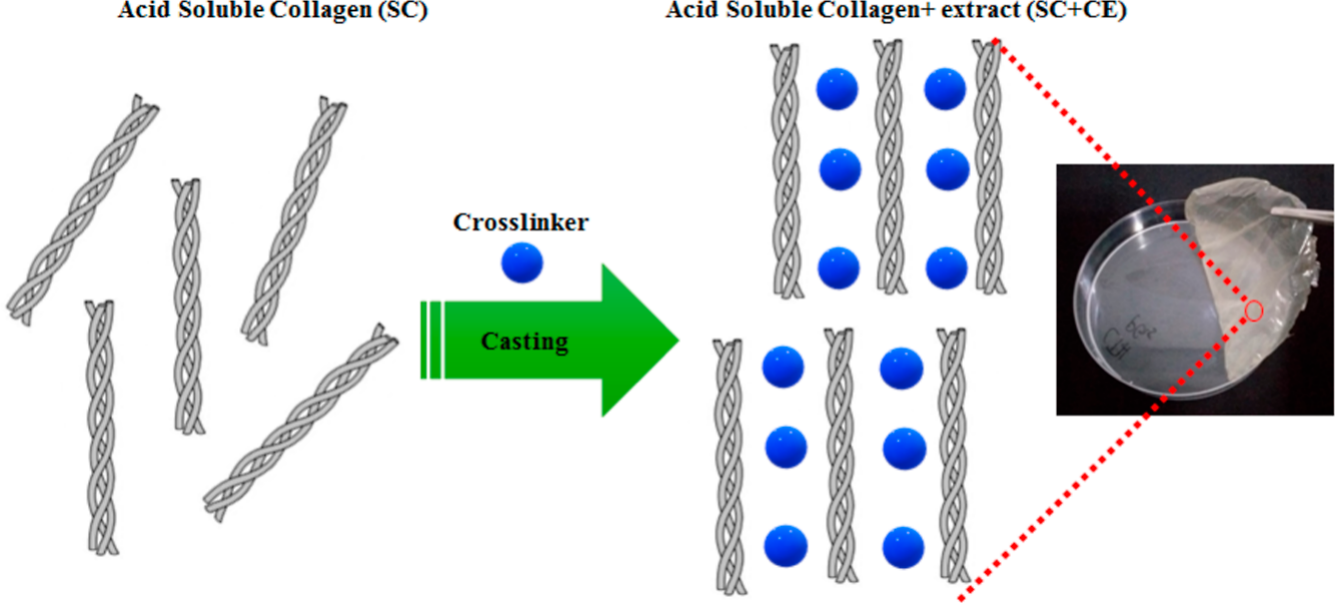
Schematic illustration of the cross-linking between SC and CE including optical image of SC-L3 showing the flexibility of SC-CE-based films.

In addition, prior to employing the film as an active material, its hue, transparency, width, and nature were all considered. As shown in Figure 2, the acid-soluble collagen (SC) type I used in the experiment provided good film quality. Indeed, the surface morphology was not affected by the type of cross-linker extract (CE), where all cross-linked films (SC-CE) appeared transparent, smooth, and flexible (Figure 3). Flexible films are essential for developing wearable sensors, biomedical equipment, implants, and drug delivery systems.^37−40^ The modification with CE tannins provided better film properties, which are due to the molecular interactions between SC and CE. Thus, all prepared films have high mechanical strength, as seen by how easily they peel off without breaking or collapsing. Indeed, collagen-pomegranate extract films were previously studied to evaluate their mechanical properties. Qu et al.^17^ and Bhuimbar et al.^41^ show that after the modification of fish skin collagen film with pomegranate polyphenols, the tensile strength of the film increased by 47.03% and provided an excellent antibacterial effect against various food-borne pathogens.

### 3.2 Morphological Study and EDS Analysis

**Figure 4:**
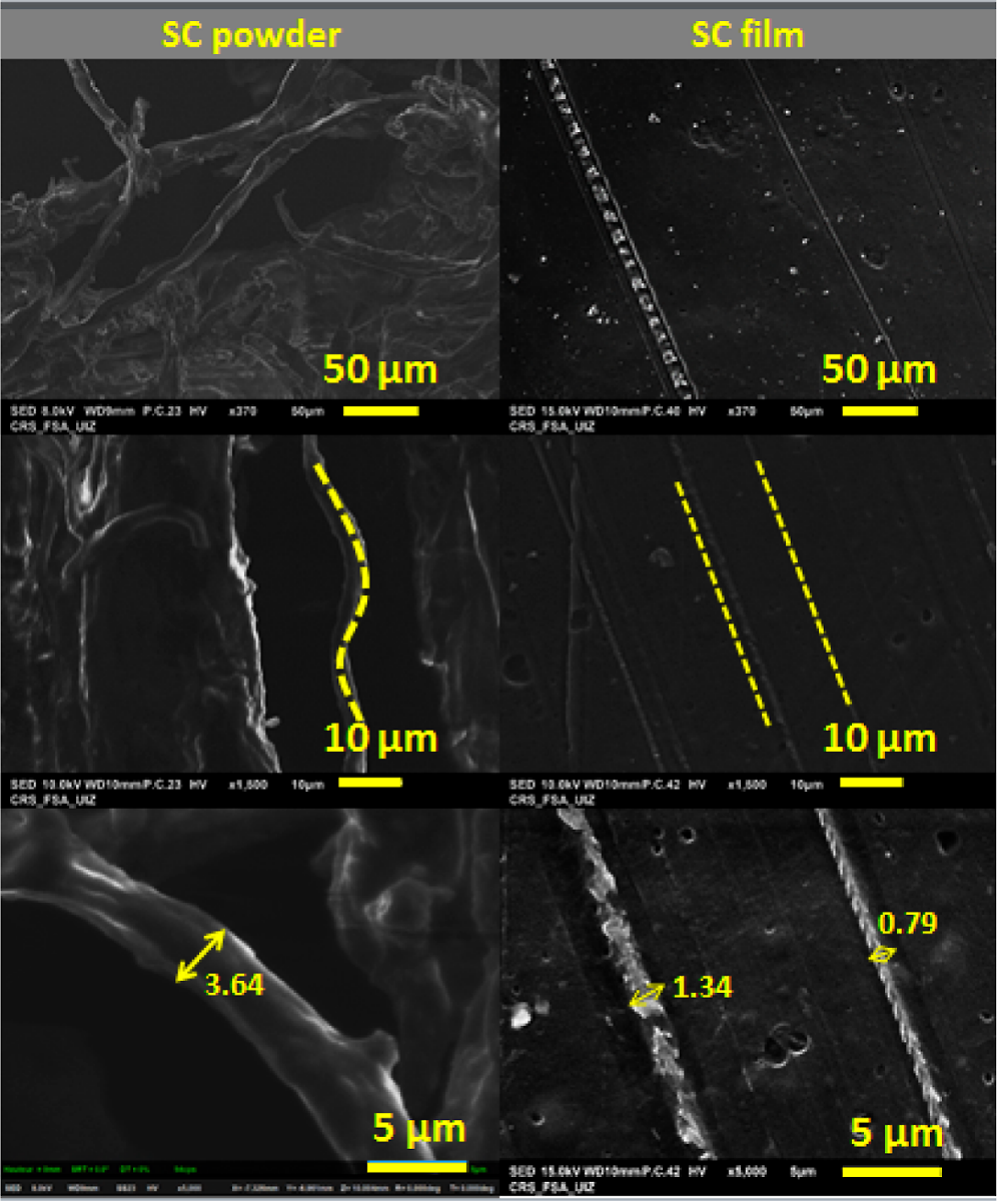
SEM comparison between SC powders and SC film micrographs.

**Figure 5:**
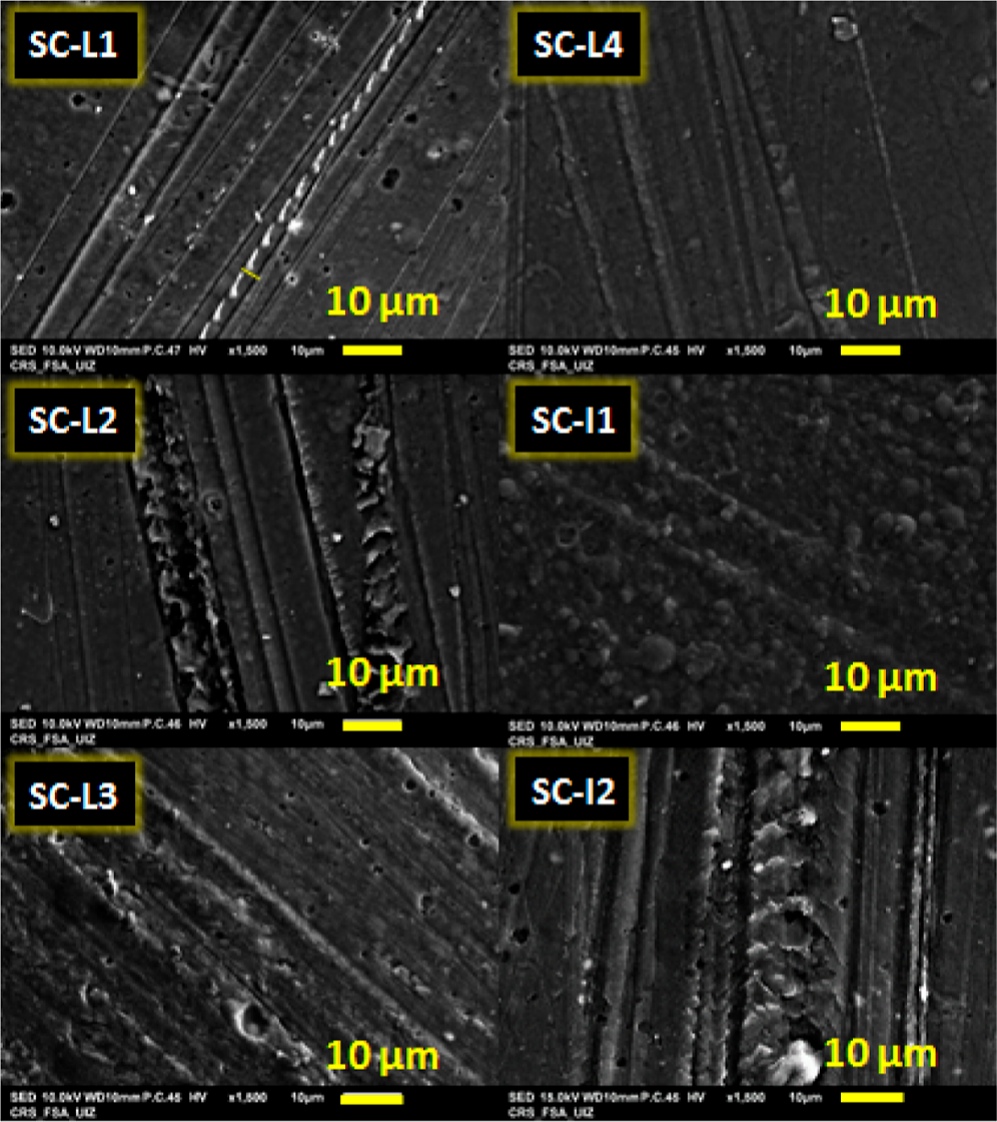
SEM micrographs of SC-L1, SC-L2, SC-L3, SC-L4, SC-I1, and SC-I2 films.

The morphology of the materials was studied via SEM before film formulation (SC powder) and after (SC film) (Figure 4). SEM images of cross-linked SC (SC-L1, SC-L2, SC-L3, SC-L4, SC-I1, and SC-I2) are shown in Figure 5.

In Figure 4, the SC powder exhibited a delicate and airy fibrous structure. The diameter of the microfibers was determined using ImageJ software. Consequently, the average fiber diameter was approximately 3.64 μm. The fibers appeared in SC more spaced out when compared to the insoluble collagen^1^ (Figure S5 in Supporting Information), which is explained by the covalent binding at the level of telopeptides on insoluble collagen molecule.^42^ On the other hand, the fibers in SC films appeared better organized and followed a straight shape. After cross-linking (Figure 5), the films show a rough surface compared to non-cross-linked SC films. The cross-linked films show a straight structure of regular interconnected fibers with a size of 2–10 μm. A study by Bertolo et al.^43^ showed that the packaging of collagen fibrils was loosening while decreasing the porosity. However, our findings differ, as we observed. The films had a smooth, compact, flattened surface, and collagen fibrils were uniformly packaged. This behavior could be explained by the saturation of the linkages of the collagen polymeric system with phenolic components with the CE concentration.^43^

**Figure 6:**
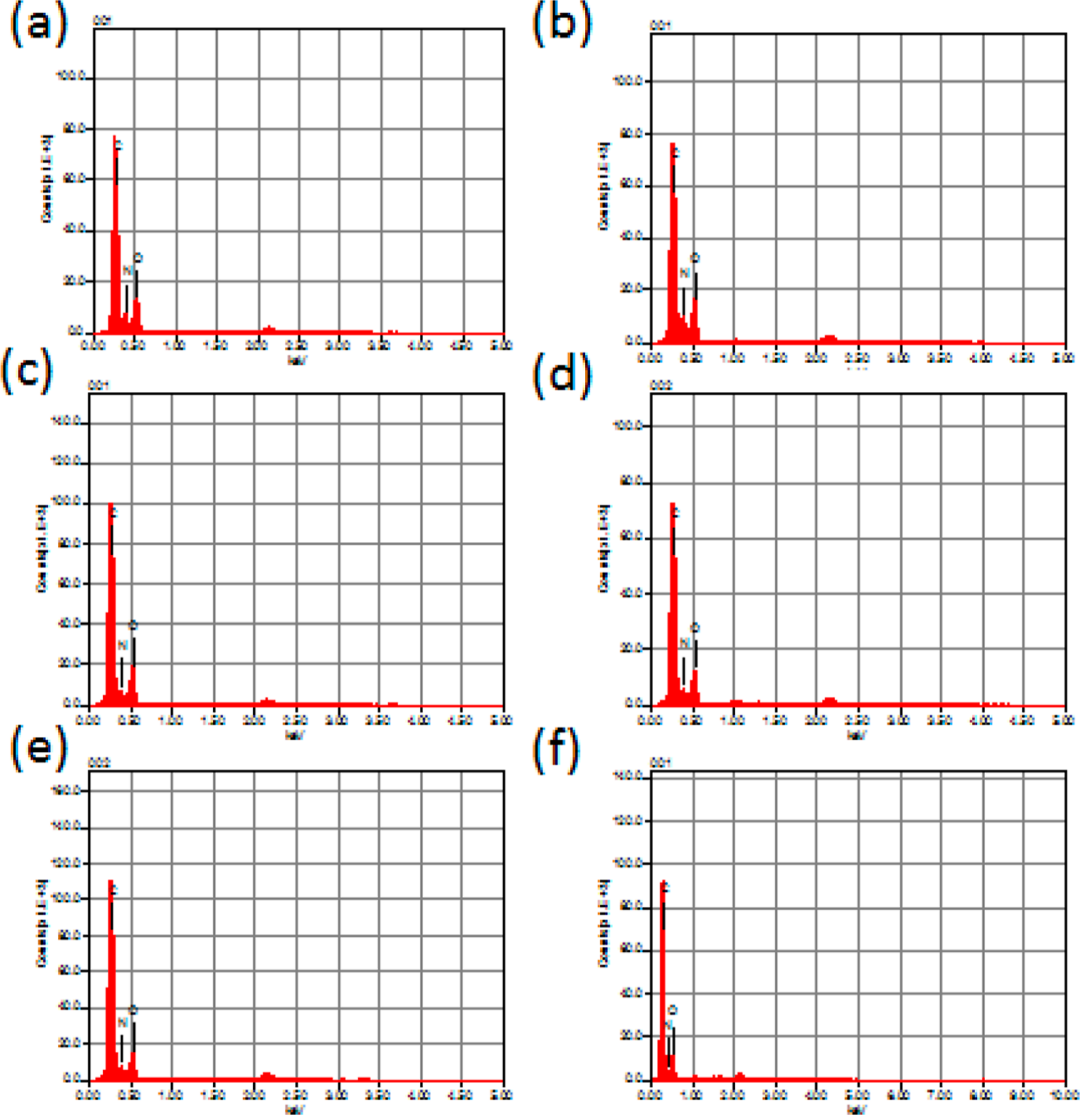
EDS analysis of IC-CE films: (a) SC-L1, (b) SC-L2, (c) SC-L3, (d) SC-L4, (e) SC-I1, and (f) SC-I2 films.

The cross-linked SC films were analyzed using EDS on three regions, all showing consistent presence of oxygen, carbon, and nitrogen (Figure 6 and Table 1). This confirmed the films as pure organic material, free from contaminants, indicating a precise and effective formulation process.

**Table 1:** EDS Analysis Outputs^a^

sample	carbon (mass %)	nitrogen (mass %)	oxygen (mass %)
SC-L1	61.83	6.94	31.23
SC-L2	57.50	7.81	34.69
SC-L3	60.77	5.19	34.04
SC-L4	61.83	5.99	32.18
SC-I1	63.12	5.73	31.15
SC-I2	65.30	3.99	30.71

a SC: soluble collagen type I; SC-CE: soluble collagen cross-linked to CE varieties (CE = L1, L2, L3, L4, I1, and I2).

### 3.3 Structural Study

**Figure 7:**
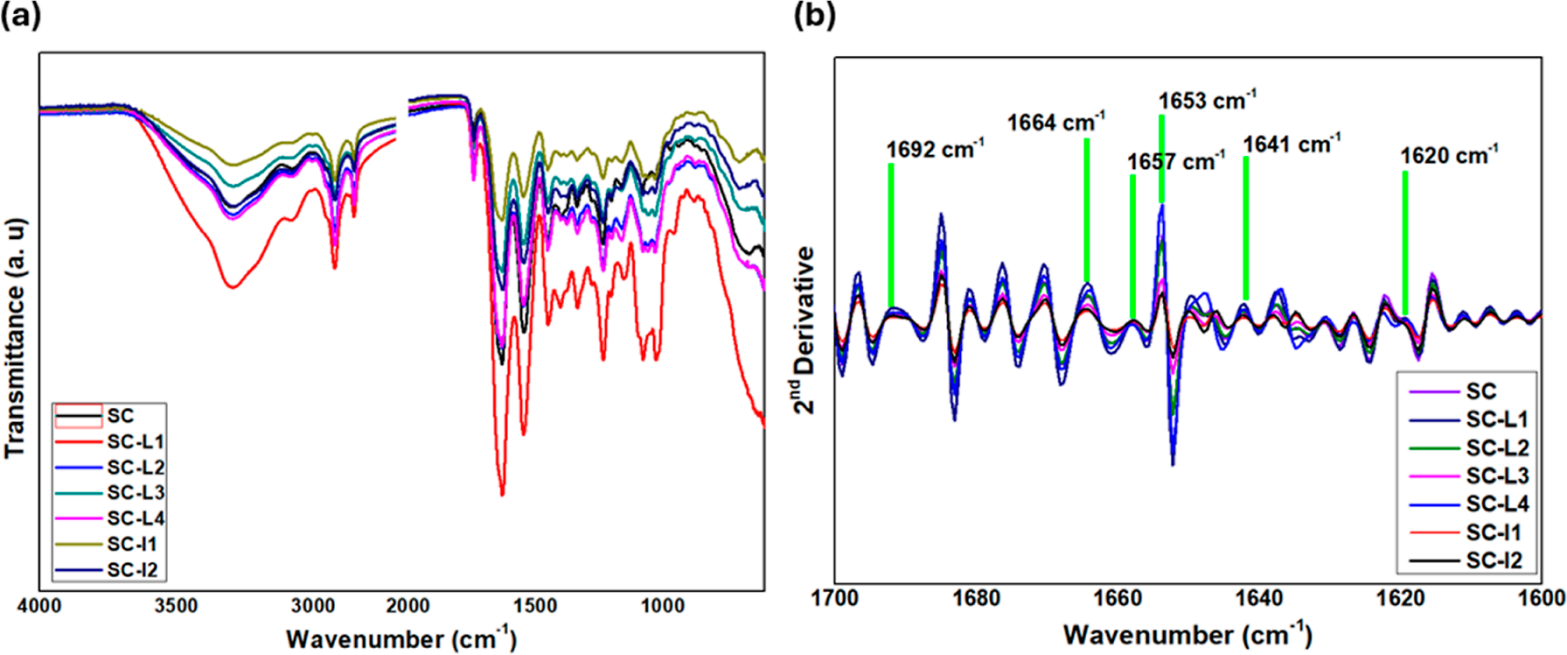
(a) FTIR spectra and (b) second derivative spectra of SC, SC-L1, SC-L2, SC-L3, SC-L4, SC-I1, and SC-I2 films.

FT-IR spectroscopy was conducted to understand the cross-linking interaction between SC films and tannins from CE extracts (L1, L2, L3, L4, I1, and I2). The infrared spectra of SC, SC-L1, SC-L2, SC-L3, SC-L4, SC-I1, and SC-I2 are depicted in the range of 600–3700 cm^–1^ in Figure 7a. The amide A band position was found in SC at 3303 cm^–1^ due to hydrogen-bonded hydroxyl groups (O–H). A shift of the amide A band to lower frequencies (3299, 3297, 3299, 3297, 3295, and 3298 cm^–1^) was observed in SC-L1, SC-L2, SC-L3, SC-L4, SC-I1, and SC-I2, which might indicate a rise in hydrogen bonding between collagen molecules.^44^ Additionally, the spectrum of SC dispersions demonstrated a characteristic pattern reflecting the amide I band at 1632 cm^–1^, the amide II band at 1548 cm^–1^, and the amide III band at 1238 cm^–1^, respectively. The amide I band, which is dominantly attributed to the stretching vibrations of peptide C–O groups, and the amide III band are linked to the secondary structure of proteins and showed that helical structure exists.^45−47^ The intensity of the carbonyl oxygen and links between amide units determine each C–O bond’s vibrational frequency; these factors are also impacted by local peptide conformation, which modifies the secondary structure.^48^ The amide I band in cross-linked SC shifted to higher frequencies (1633–1634 cm^–1^), which suggested that collagen molecules were reinforced by covalent cross-links. Table 2 represents all the vibrational wavenumber values of cross-linked collagen films by the six extracts (SC-CE) compared to SC. Figure 7b demonstrates that the positions of amides I and II, related to the collagen triple helix, do not change with the change of the extract variety (CE) and are still maintained at the same wavenumber unless other amid bands (A, B, III) are broadened to some degrees. According to He et al.,^49^ once condensed tannin (e.g., procyanidin) extracts were added, the collagen triple helix structure in the films may still be retained.^49^ It is conceivable that tannins from CE might form new hydrogen bonds with the collagen (FTIR spectra of CE varieties are represented in Figure S6 of the Supporting Information). Collagen’s helix structure is retained as a result. In other words, PR tannins have a cross-linking effect without damaging collagen’s matrix. Moreover, the peak intensities confirm that extracts behave differently with functional groups of collagen.^1^ There is a strong consensus that plant tannins stabilize collagen in acidic environments primarily through hydrogen bonding. Minor differences in the dipole properties of phenolic compounds can impact their binding to collagen. The hydroxyl, carboxyl, amino, and amide groups in collagen side chains are potential sites for forming hydrogen bonds with CE phenolic hydroxyl groups.^49^

**Table 2:** Different Wavenumber Peaks of SC before and after Cross-Linking^a^

sample	wavenumber peaks (cm^–1^)					
	amide A	amide B	C=O	amide I	amide II	amide III
SC	3303	2924	1746	1632	1548	1238
SC-L1	3299	2926	1745	1634	1546	1234
SC-L2	3297	2926	1745	1633	1546	1237
SC-L3	3299	2924	1745	1634	1546	1236
SC-L4	3297	2926	1744	1634	1546	1235
SC-I1	3295	2925	1745	1634	1546	1240
SC-I2	3298	2926	1745	1633	1545	1238

a SC: soluble collagen type I; SC-CE: soluble collagen cross-linked to CE varieties (CE = L1, L2, L3, L4, I1, and I2).

To get more information about protein structure, a second derivative of FTIR spectra was carried out in the range of 1600–1700 cm as conducted by Venezia et al.^50^Figure 7b shows the results of the second derivative provided by the Origin software. Table 3 represents the band assignment of amide I which contains six peaks at 1692, 1664, 1657, 1653, 1653, 1641, and 1620 characteristics to intermolecular bonds, triple helix, α-helix, and β-sheets.

**Table 3:** Assignments of Second Derivative Spectra

wavenumber peaks (cm^–1^)	functional groups
1692	intermolecular bonds
1664	triple helix
1657	triple helix
1653	α-helix
1641	α-helix
1620	intermolecular bonds and β-sheets

### 3.4 Thermal Behavior Study

**Figure 8:**
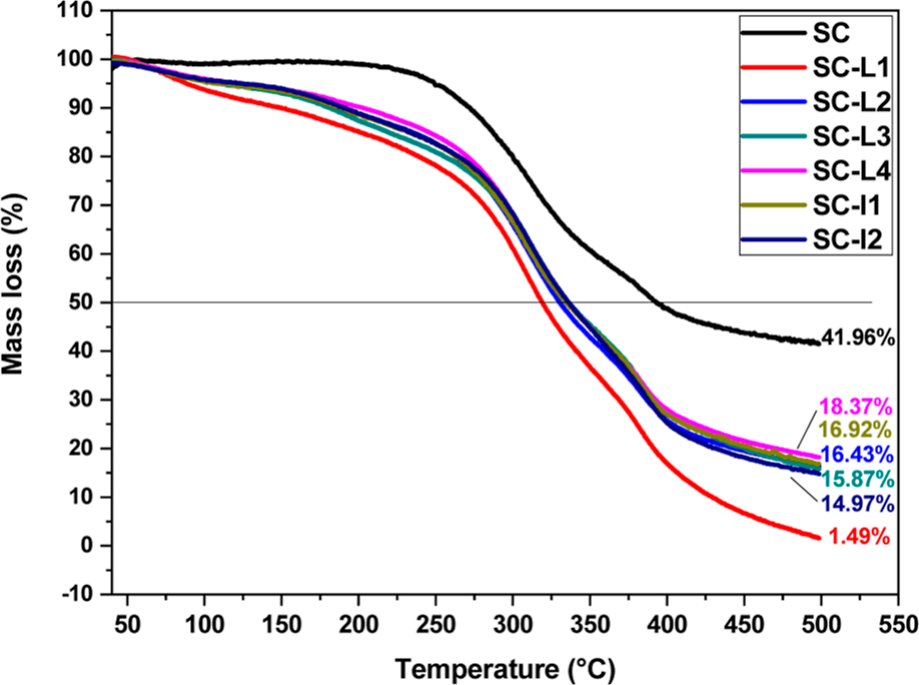
TG analysis of SC, SC-L1, SC-L2, SC-L3, SC-L4, SC-I1, and SC-I2 film.

The thermal stabilities of SC-L1, SC-L2, SC-L3, SC-L4, SC-I1, and SC-I2 films were carried out from room temperature to 500 °C. This study aimed to evaluate the thermal resistance of cross-linked films and to understand the effect of CE varieties through thermal stabilities. As shown in Figure 8, the TGA profiles of SC and cross-linked SC-CE are comparable. There were typically three stages of weight loss found. The first stage below 100 °C is attributed to the breakage of inter- and intramolecular hydrogen bonds associated with a gradual water loss.^51^ The cross-linked SC films have shown high dehydration when compared to non-cross-linked ones. This can be attributed to the easiest evaporation of physisorbed water of SC film in the preparation process during the drying step under ventilation; water retention in the collagen triple helix is expected to be significant when the latter is cross-linked to sugars.^52^ Water molecules can be attracted to polar C–O bonds of sugars contained in CE interacting with SC, which explains the high water retention of SC-CE films compared to non-cross-linked SC. The second stage, between 250 and 350 °C, is attributed to the decomposition of the collagen chains, where 40% of weight loss occurred for cross-linked and non-cross-linked films due to the larger macromolecules fragmentation into smaller ones. The third maximum weight loss was observed beyond 350 °C for SC and SC-CE films, indicating the production of gaseous elements. Similar patterns have been reported in earlier studies, with major degradation occurring at 500 °C.^53,54^

In order to evaluate the influence of cross-linker sources, all cross-linked SC are compared with uncross-linked one. For this, the temperature at 50% (*T*° 50) weight losses and the residues (%) at 500 °C (*R* % 500) were calculated (Table 4). *T*° 50 were ordered as follows: SC-I2 > SC-L3 > SC-L4 > SC-I1 > SC-L2 > SC-L1. The residues (%) were ordered as follows: SC-L4 > SC-I1 > SC-L2 > SC-L3 > SC-I2 > SC-L1. The hydrogen bonds that bind the tropocollagen helix are disrupted when collagen is heated; the molecule loses its fibrillary structure, and it takes on a random coil form. Adding cross-links with PR tannins affects the thermal stability of collagen structure. However, as can be seen from Table 4, the weight losses of cross-linked films increased by around 20% compared to non-cross-linked ones (SC), showing that the thermal denaturation and degradation are catalyzed by the cross-linking with CE. Similar results were reported by Kumar et al.^55^ on chitosan films cross-linked to pomegranate peel extract, where a higher concentration of the latter had significantly (*p* < 0.05) decreased film transparency, solubility, swelling, and color, as well as the thermal stability. SC fibril structures are not bridged by covalent linkage of telopeptides (case of insoluble collagen),^56^ allowing CE tannins to be integrated more in the interfibrillary distances. The decrease in stability could be explained by the hydrogen bonding sites of water provided by CE tannins, where a stable network structure formation is promoted within the film and stops water evaporation. Consequently, after the dehydration, the triple helix amount and structure with vacant sites of water become more affected by the pyrolysis temperature that hydrolyzed the peptide bonds.^57,58^ Compared to the insoluble collagen stability, the latter could be attributed to intermolecular hydrogen bonding between free hydroxyl groups of hydroxyproline and CE tannins on the surface of collagen fibril that is more susceptible to interact with cross-linkers than the interfibrillar space bridged by the lysine and hydroxylysine covalent linkages.^59^ This surface cross-linking process could ultimately protect the fibril from the action of heat.

**Table 4:** TGA Outputs^a^

samples	temperature at 50% loss % (°C)	residue (%) at 500 °C
SC	392.41	41.96
SC-L1	318.64	1.49
SC-L2	329.75	16.43
SC-L3	336.06	15.87
SC-L4	334.93	18.37
SC-I1	333.92	16.92
SC-I2	336.32	14.97

a SC: soluble collagen type I; SC-CE: soluble collagen cross-linked to CE varieties (CE = L1, L2, L3, L4, I1, and I2).

### 3.5 Nanoscaled Observation Using TEM

**Figure 9:**
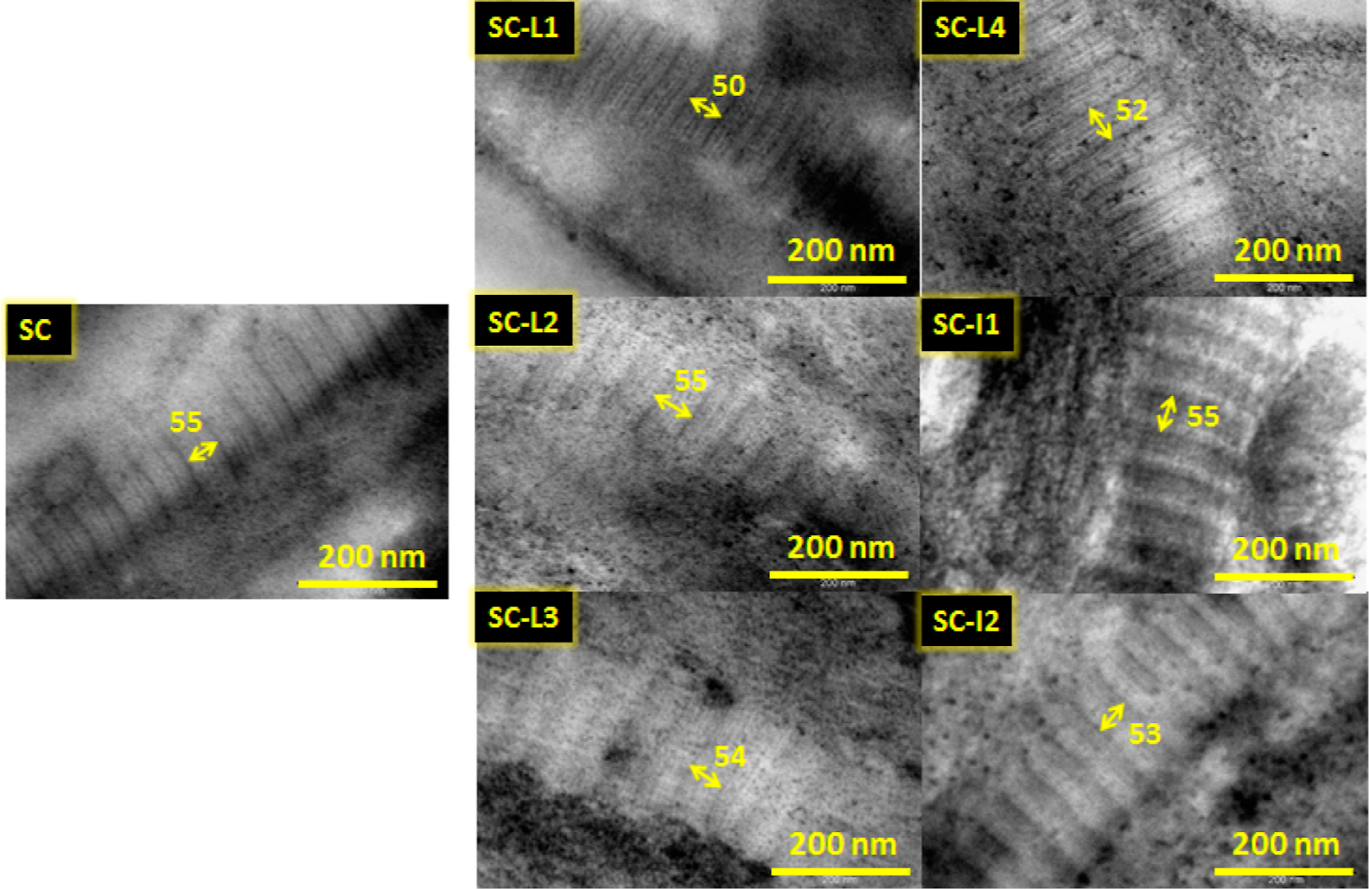
TEM images of SC, SC-L1, SC-L2, SC-L3, SC-L4, SC-I1, and SC-I2 films.

TEM analysis is crucial for exhibiting the nanoscaled structures of SC films before and after cross-linking. Figure 9 shows that well-ordered collagen fibrils illustrated by dark–light periodic patterns are observed with different D-periodic fibrils. Indeed, D-periods are typically in the range of 50–55 nm where each one is assembled by a Gap (Light period) and an Overlap (Dark period) sequences that measure depending on the intermolecular cross-linking.^60^ From the measures taken by ImageJ software, the Overlap periods ranged from 4.8 nm for non-cross-linked SC to 32 nm for SC-I1 (Table 5). SC-I1 and SC-I2 showed the highest overlap periods, meaning a high cross-linking density when using I1 and I2 cross-linker extracts. The overlap periods were ordered as follows: SC-I1 > SC-I2> SC-L3> SC-L2> SC-L4> SC-L1.

**Table 5:** D-Periods Were Measured from TEM Images of SC Films^a^

SC film	D-period (nm)	overlap (nm)	fibril width (nm)
SC	54.73 ± 1.67	4.89 ± 0.28	182.52 ± 4.49
SC-L1	50.03 ± 3.84	5.61 ± 0.56	159 ± 3.87
SC-L2	54.6 ± 1.48	6.2 ± 0.48	159.88 ± 7.82
SC-L3	54.43 ± 3.13	7.41 ± 1.27	200.28 ± 6.38
SC-L4	52.35 ± 0.18	5.81 ± 0.62	196.17 ± 9.57
SC-I1	55.06 ± 1.01	**32.38±2.37**	177.15 ± 5.37
SC-I2	53.04 ± 0.44	**25.19±0.79**	176.09 ± 2.77

a SC: soluble collagen type I film; SC-CE: soluble collagen cross-linked to CE varieties (CE = L1, L2, L3, L4, I1 and I2).

In the micrographs of SC treated with CE tannins, a higher order of fiber splitting is observed as reported earlier.^61^ It can be clearly seen that films modified with L3 and I2 bring a higher degree of orderliness in the fiber packing. This is supported by their higher temperature at 50% of loss observed in TGA analysis where the increase can be described by fiber cross-linking and long-range ordering.^61,62^

## 4 Conclusions and Perspectives

This research work aimed to develop a novel cross-linked film that depicts a promising future in food or medical applications and promote pomegranate byproduct valorization. The studies on the structure and thermal behavior of collagen films in the presence of PR tannins can lead to the following conclusions. The obtained SC-CE cross-linked films were translucent, thin, and light yellow in color. Through FTIR spectroscopy, the observable changes in their physicochemical characteristics were reported. Through SEM examination, the variation in the internal morphology (porosity and fiber organization) of the cross-linked SC films was examined. Using TGA analysis, we also investigated the thermal property. The thermal degradation shows that more than 80% of the initial film weight was lost at 500 °C; an activation of degradation after cross-linking to CE was detected. Further, it was noticed that SC-I1 and SC-I2 films exhibited higher cross-linking density, which was detected by the TEM analysis. However, the SC films dedicated to biomedical applications should focus more on good biocompatibility and low toxicity, which is taken as a major concern. Further studies should be focused on studying SC-CE films’ ability to serve as grafts in medical applications or as a coating for active food packaging.

## Supporting Information Available

Additional characterization details, including chromatograms and FTIR vibrational spectra of CE varieties, in addition to supplementary morphological and structural characterizations of insoluble collagen type I (PDF)

## Abbreviations

CE: cross-linker extract
FTIR: Fourier transform infrared spectroscopy
PR: pomegranate rind
*R* %_500_: residue (%) at 500 °C
SC: acid-soluble collagen
SC-CE: cross-linked films
SEM: scanning electron microscopy
*T*° 50: temperature at 50% loss % (°C)
TEM: transmission electron microscopy
TGA: thermogravimetric analysis
