# Supplementary material for: Sustainable Collagen Film Preparation with Tannins Extracted from Moroccan Pomegranate Byproduct Varieties: Thermal, Structural, and Nanoscaled Studies
Source: ACS Omega. 2024 Jun 12;9(25):27428–37. doi: 10.1021/acsomega.4c02321 (PMC11209680; doi:10.1021/acsomega.4c02321)
Supplement: Supplementary file 1 — ao4c02321_si_001.pdf [file ao4c02321_si_001.pdf]

Sustainable collagen films preparation with tannins extracted from Moroccan pomegranate byproducts varieties: thermal, structural and nanoscaled studies

Sara El Moujahed[1], Faouzi Errachidi[2], Ana-Maria Morosanu[3], Hicham Abou Oualid[4], Sorin Marius Avramescu[5], Mihaela Dragoi Cudalbeanu[6], Fouad Ouazzani Chahdi[1], Youssef Kandri Rodi[1] and Rodica-Mihaela Dinica[7].

[1]*Laboratory of Applied Organic Chemistry, Faculty of Sciences and Technologies, Sidi Mohamed Ben Abdellah University, Fez, Morocco;*
[2]*Laboratory of Functional Ecology and Engineering Environment, Faculty of Sciences and Technologies, Sidi Mohamed Ben Abdellah University, Fez, Morocco;*
[3]*Institute of Biology Bucharest, Romanian Academy, Bucharest, Romania;*
[4]*Green Energy Park, IRESEN-UM6P, Benguerir, Morocco;*
[5]*Department of Organic Chemistry, Biochemistry and Catalysis, Faculty of Chemistry, University of Bucharest, Bucharest, Romania;*
[6] *Faculty of Land Reclamation and Environmental Engineering, University of Agronomic Sciences and Veterinary Medicine of Bucharest, Bucharest, Romania*
[7]*Laboratory of Organic Chemistry, Faculty of Sciences and Environment, Dunarea de Jos University of Galati, Galati, Romania;*
**Correspondence: sara.elmoujahed@gmail.com*

Supporting Information

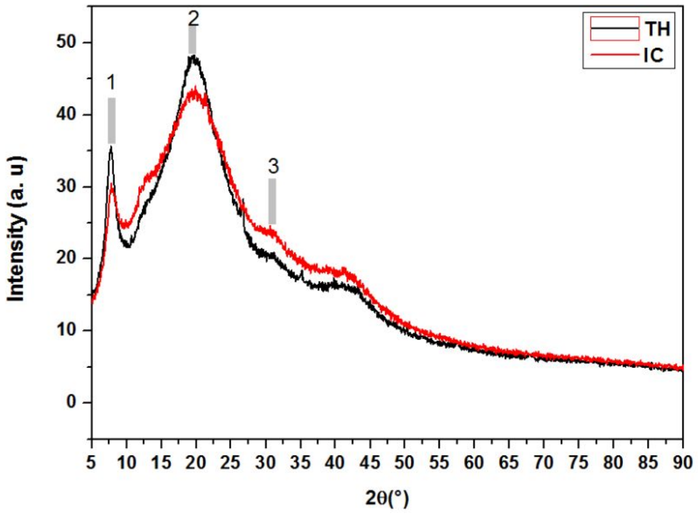

Figure S1. XRD patterns of insoluble collagen (IC) compared to acid-soluble collagen (TH) type I.

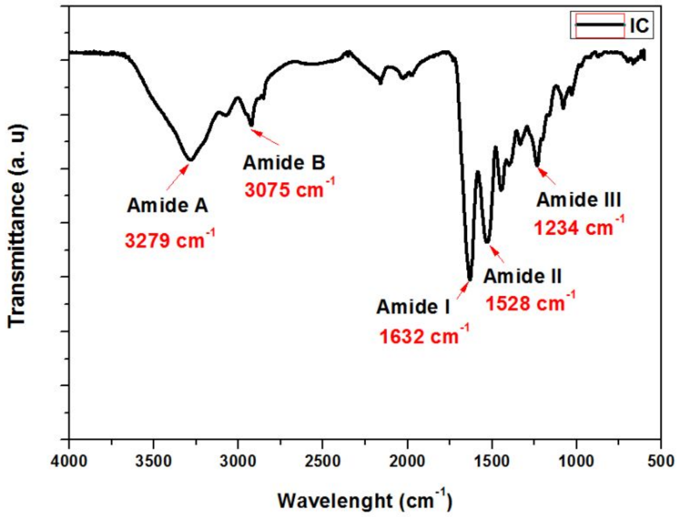

Figure S2. FTIR vibrational spectrum of collagen type I (Insoluble and Acid-soluble).

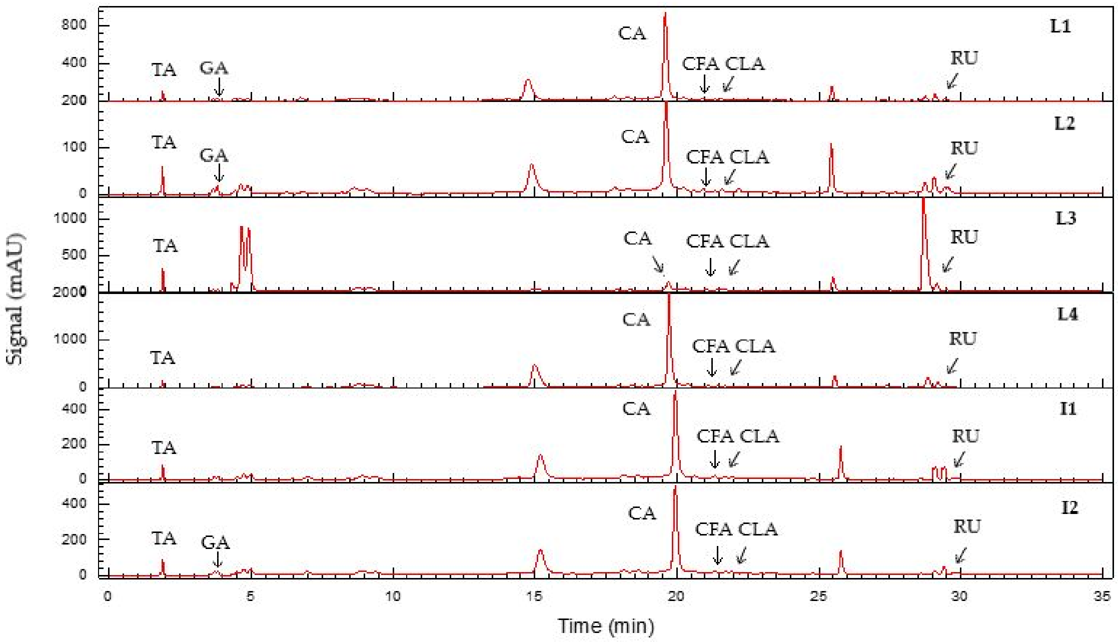

Figure S3. HPLC-DAD chromatograms of L1, L2, L3, L4, I1, I2 PR extracts. TA - tannic acid; GA - gallic acid; CFA - Caffeic acid; CLA - Chlorogenic acid; CA - Catechin; RU – Rutin recorded at 250 nm.

Table S1. Quantification data of identified CE varieties phenolic compounds by λmax each standard compound by HPLC-DAD analysis.

Compound name	T_R (min)	Concentration (mg/kg dw)					
		**L1**	**L2**	**L3**	**L4**	**I1**	**I2**
**Tannic acid**	1.90	0.35	0.26	0.54	0.46	0.39	0.38
**Gallic acid**	3.77	0.58	0.39	ND	ND	ND	0.63
**(+) Catechin**	19.60	**690.84**	**115.79**	**93.63**	**1579.91**	**486.88**	**403.65**
**Caffeic acid**	20.25	0.10	0.12	0.23	0.27	0.20	0.19
**Chlorogenic acid**	20.93	1.74	0.52	0.96	1.19	1.91	1.20
**Rutin**	29.10	2.41	1.11	3.19	4.29	3.28	1.08

ND – not determined. Retention time (T_R) error of mean for compounds was ± 0.0001–0.2 min. Data were expressed as mg/kg on a dry weight basis.

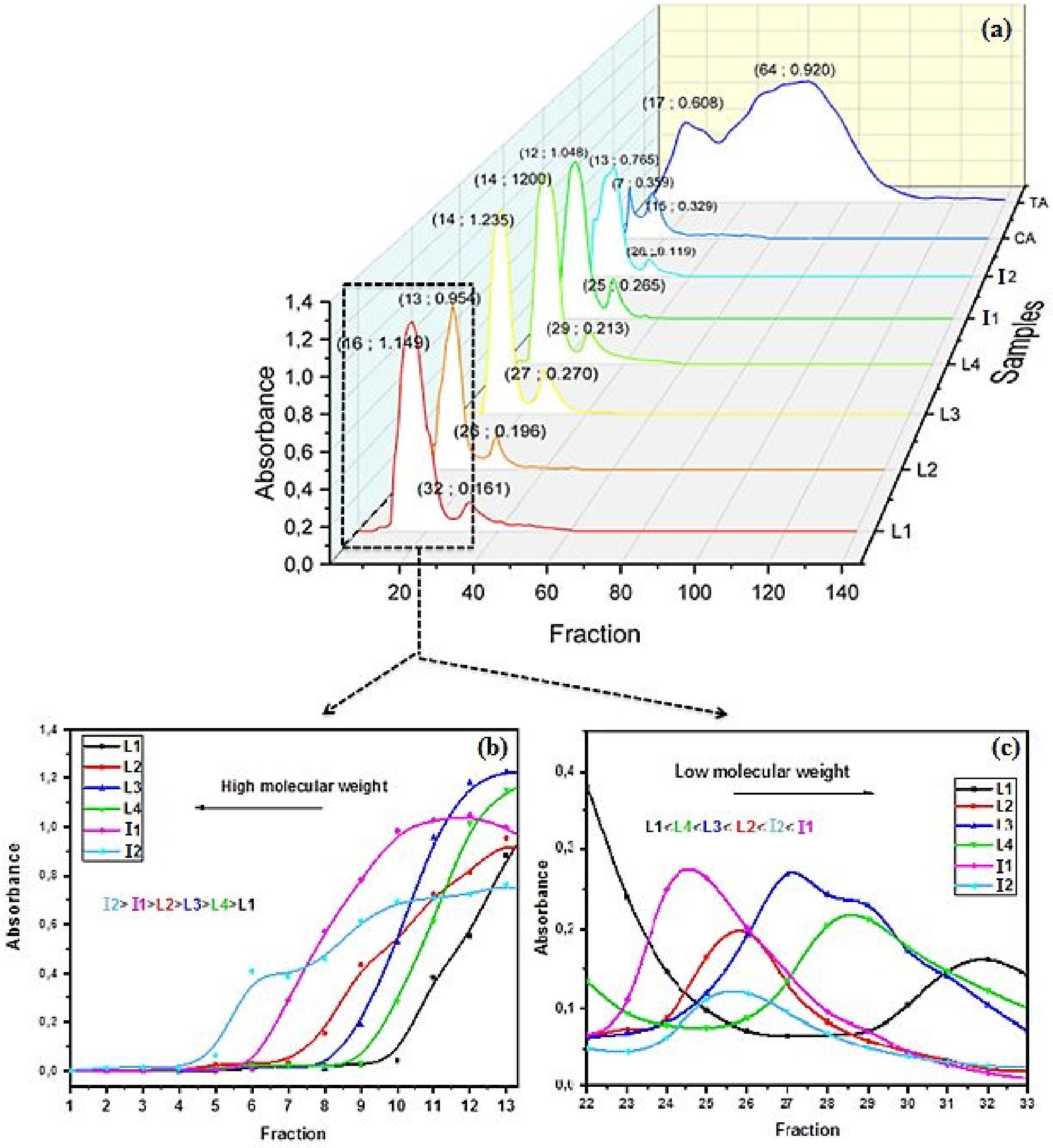

Figure S4. (a) Comparative representation of elution profiles on Sephadex G-50 crosslinkers extracts (L1, L2, L3, L4, I1 and I2) from studied PR varieties; (b) Zoom on high molecular weight distribution; (c) Zoom on low molecular weight distribution of crosslinkers extracts (CE).

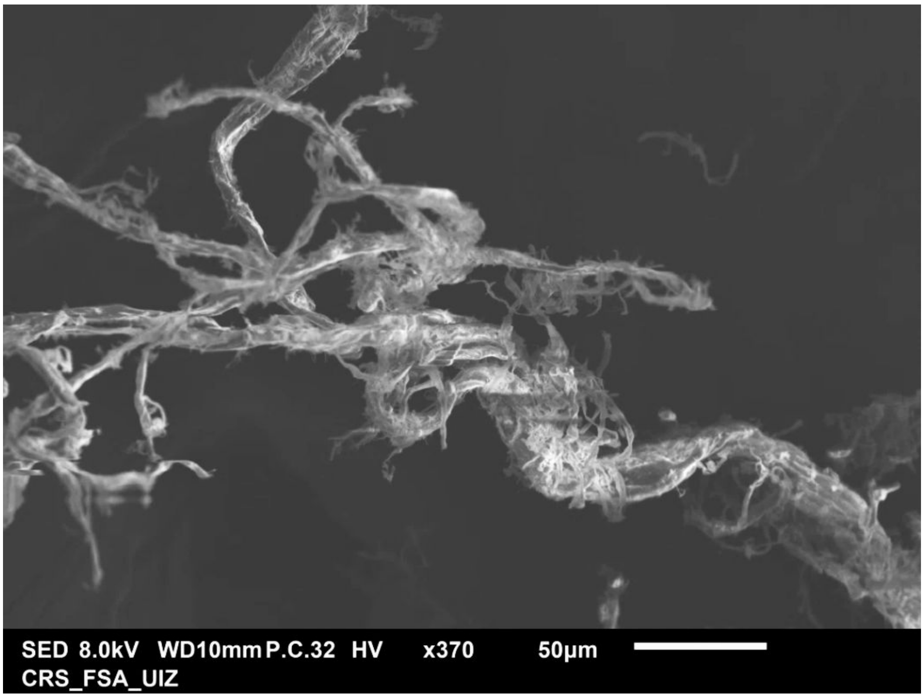

Figure S5. SEM micrograph of insoluble collagen type I.

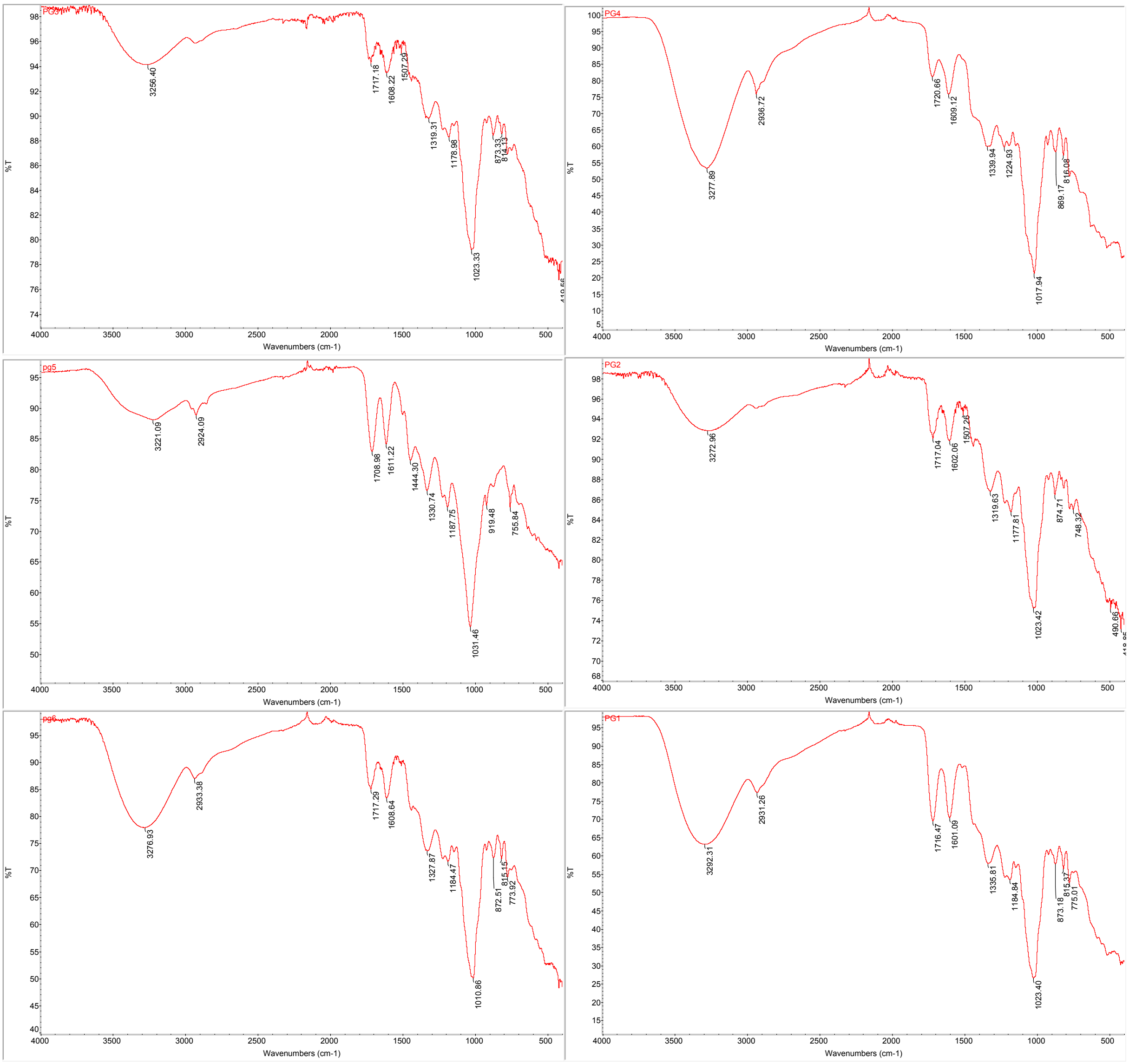

Figure S6. FTIR of L1, L2, L3, l4, I1 and I2 extracts
